# Bioenergetic State of Escherichia coli Controls Aminoglycoside Susceptibility

**DOI:** 10.1128/mbio.03302-22

**Published:** 2023-01-10

**Authors:** Jessica Y. El Khoury, Jordi Zamarreño Beas, Allison Huguenot, Béatrice Py, Frédéric Barras

**Affiliations:** a Département de Microbiologie, SAMe Unit, Institut Pasteur, Paris, France; b Université Paris-Cité, Paris, France; c UMR CNRS 6047, Paris, France; d Laboratoire de Chimie Bactérienne UMR7283, Centre National de la Recherche, Marseille, France; e Aix-Marseille Université, Marseille, France; f Institut de Microbiologie de la Méditerranée, Marseille, France; g Institut Microbiologie Bioénergies et Biotechnologie, Marseille, France; Racah Institute of Physics, Hebrew University of Jerusalem

**Keywords:** *Escherichia coli*, aminoglycosides, bioenergetics, fumarate respiration, proton motive force, RavA-ViaA

## Abstract

Aminoglycosides (AG) have been used against Gram-negative bacteria for decades. Yet, how bacterial metabolism and environmental conditions modify AG toxicity is poorly understood. Here, we show that the level of AG susceptibility varies depending on the nature of the respiratory chain that Escherichia coli uses for growth, i.e., oxygen, nitrate, or fumarate. We show that all components of the fumarate respiratory chain, namely, hydrogenases 2 and 3, the formate hydrogenlyase complex, menaquinone, and fumarate reductase are required for AG-mediated killing under fumarate respiratory conditions. In addition, we show that the AAA+ ATPase RavA and its Von Wildebrand domain-containing partner, ViaA, are essential for AG to act under fumarate respiratory conditions. This effect was true for all AG that were tested but not for antibiotics from other classes. In addition, we show that the sensitizing effect of RavA-ViaA is due to increased gentamicin uptake in a proton motive force-dependent manner. Interestingly, the sensitizing effect of RavA-ViaA was prominent in poor energy conservation conditions, i.e., with fumarate, but dispensable under high energy conservation conditions, i.e., in the presence of nitrate or oxygen. We propose that RavA-ViaA can facilitate uptake of AG across the membrane in low-energy cellular states.

## INTRODUCTION

Antibiotic resistance is an important biomedical and societal problem, challenging the ability to treat bacterial infections (1). Modulation of intracellular antibiotic concentrations is one of the most common processes leading to resistance, either by limiting antibiotic entry, or by increasing efflux (2). Among the reasons antimicrobials may not cure infections as efficiently as often hoped are environmental conditions that can interfere with uptake of the antibiotic. For example, the low pH and anaerobic environment of an abscess is particularly deleterious to the efficacy of aminoglycosides (AG) (3, 4). In addition, antibiotic tolerance, defined as the ability of bacteria to tolerate an antibiotic without affecting its MIC value, plays an important role in the evolution of antibiotic-resistant cells (5). Several studies have emphasized the importance of bacterial metabolism in modulating the level of tolerance (6), and it has been shown that the conversion of tolerant cells to susceptible cells can be achieved by the addition of exogenous metabolites, such as amino acids, tricarboxylic acid cycle (TCA) metabolites, or nucleotides (7). Moreover, our previous work revealed how redox cyclic drugs antagonize fluoroquinolones and how iron limitation can lead to an increased level of tolerance to AGs (8–10).

AGs are commonly used worldwide due to their high efficacy and low cost. AGs include kanamycin, tobramycin, gentamicin (Gm), neomycin, amikacin, and streptomycin. This class of antibiotics targets the ribosome, resulting in mistranslation and ultimately cell death. Uptake of AG begins with the entry of a small amount of the antibiotic through proton motive force (*pmf*)-dependent mechanisms (11–13) or other transport systems (14–16) initiating mistranslation by the ribosome, which leads to membrane damage by incorporation of mistranslated proteins and a second wave of massive AG uptake (11, 17). The *pmf* is particularly important during the first phase of uptake (18), whereas the second phase occurs in response to defective translation. The *pmf* is produced by the activity of respiratory complexes, and we have previously shown that the maturation of respiratory complexes has a direct impact on the efficiency of AG uptake and the level of susceptibility (8). One prediction is that the more energetic the respiratory chain used, the higher the *pmf* produced and the more potent the AG-mediated killing. This simple view is reminiscent of an early suggestion that Gm transport may be related to the level of the membrane potential (Δψ) (3).

E. coli contains a variety of branched respiratory chains. These chains consist of dehydrogenases oxidizing different substrates acting as electron donors (formate, H_2_, NADH, glycerol-3-phosphate, etc.). Electrons are then transferred to terminal reductases via ubiquinol (UQ) or naphthoquinols (menaquinol [MKH_2_] and demethylmenaquinol [DMKH_2_]). A wide range of compounds can then be used as final electron acceptors, such as oxygen (O_2_), nitrate (NO_3_^–^), fumarate, or trimethylamine N-oxide (TMAO) (19). When considering respiration in E. coli, energy conservation is maximal with O_2_ and minimal with fumarate. Synthesis of hydrogenases and terminal reductases depends on the identity of the electron acceptor present in the medium, and genetic regulation allows E. coli to favor respiratory chains with high ATP or growth yields. Thus, O_2_-, NO_3_^–^-, and fumarate respiratory chains are preferentially used in this order (20).

The terminal reductase Frd (fumarate reductase) allows E. coli to use fumarate as a terminal electron acceptor. Frd is composed of four subunits, including the cytosolic soluble FrdA and FrdB and the membrane subunits FrdC and FrdD. FrdB harbors a flavin adenine dinucleotide (FAD) and FrdA harbors three iron-sulfur (Fe-S) cluster cofactors. FrdB is a menaquinone (MK) oxidoreductase, which relays the electrons from MKH_2_ to the active site of FrdA that reduces fumarate to succinate (21). During fumarate respiration, Frd utilizes electrons transmitted by hydrogenase-2 (Hyd-2), glycerol-3-phosphate dehydrogenase (GlpABC) or NADH dehydrogenase I (Nuo) via MK or demethylmenaquinone (DMK) (19). It has been proposed that the multistep assembly process of Frd is under the influence of the RavA-ViaA complex formed by the AAA+ ATPase regulatory variant A (RavA) and its von Willebrand factor type A (VWA)-containing partner ViaA (22). Interestingly, evidence for a link between the function of RavA-ViaA complex and susceptibility to sublethal concentrations of AG has been reported in both E. coli and Vibrio cholerae (23–25).

In the present work, we conducted a thorough and comprehensive investigation of the relationship between the respiratory metabolism utilized by E. coli and its susceptibility to AG. We found a direct link between energy conservation and AG susceptibility level. Under fumarate respiration conditions, we highlighted the significant contribution of hydrogenase-2 (Hyd-2), formate hydrogenlyase (FHL) complex, and fumarate reductase to this susceptibility. Furthermore, our study establishes that RavA and ViaA are enhancers of AG potency under anaerobic conditions. The importance of RavA-ViaA in mediating AG potency varies with the nature of the respiratory electron acceptor and we propose that RavA-ViaA links the energetic status of the cell and AG uptake.

## RESULTS

### Decreased susceptibility of E. coli to Gm under anaerobic conditions.

To investigate whether the type of metabolism used for growth influences the level of AG susceptibility, we measured MIC of the wild type (WT) strain in aerobiosis and anaerobiosis, in Luria-Bertani (LB) medium supplemented with fumarate, NO_3_^–^, or no electron acceptor, where in the latter instance we assumed mixed-acid fermentation was used (26). Under all anaerobic growth conditions tested, the MIC value of Gm was 4-fold higher than under oxygen, i.e., 8 μg/mL.

**FIG 1:**
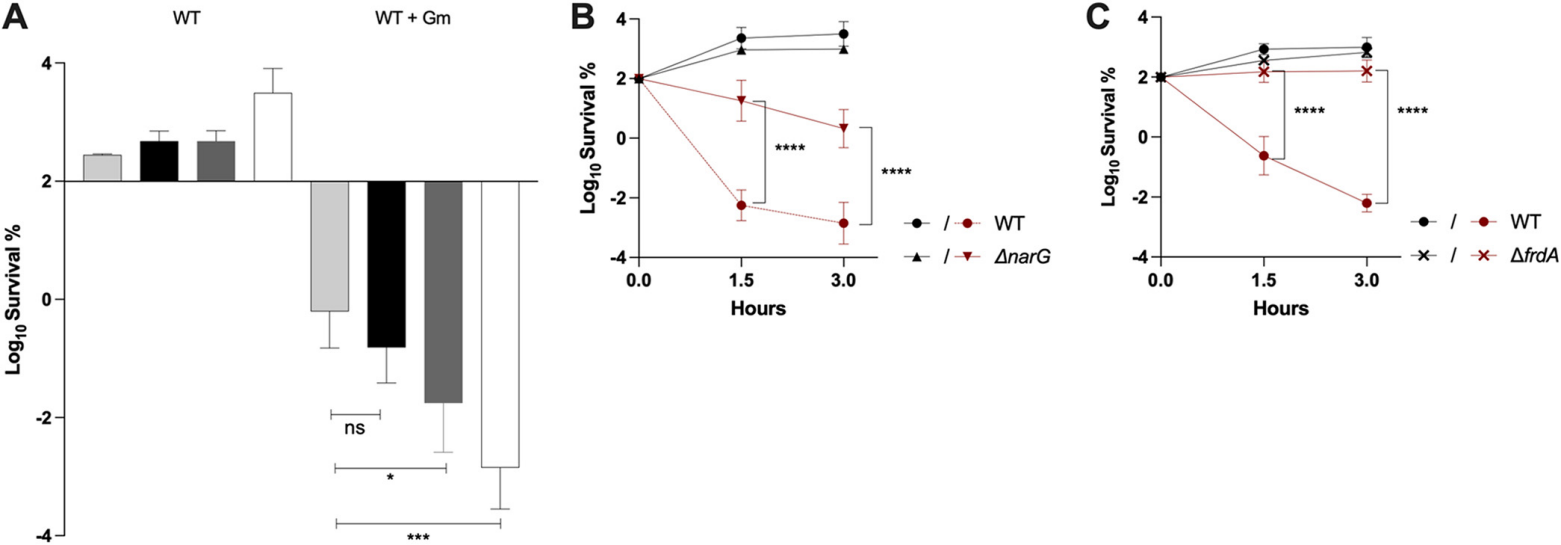
Decreased susceptibility of E. coli to Gm under anaerobia. (A) WT (FBE051) strain was grown anaerobically in LB (light gray), LB supplemented with glycerol at 0.2% (black), fumarate (dark gray) or NO_3_^–^ (white) at 10 mM. Histograms show the survival values of untreated (WT) and Gm-treated (WT + Gm) cells after 3 h. (B) Survival of WT and Δ*narG* (FBE829) strains in LB supplemented with 0.2% glycerol and nitrate at 10 mM. (C) Survival of WT and Δ*frdA* (FBE790) strains in LB supplemented with fumarate at 10 mM. (B and C) The survival values after 1.5 and 3 h of treatment are represented. Black and red lines are for untreated and Gm-treated bacteria, respectively. Survival, measured by CFU per mL, was normalized relative to time zero at which Gm was added (early log phase cells; ~5 × 10^7^ CFU/mL) and plotted as Log_10_ of survival percentage. Values are expressed as means of at least three biological replicates and error bars depict standard deviation. One-way ANOVA tests followed by Sidak’s multiple-comparison tests were performed to compare at each time point (1.5 and 3 h) the treated WT to the treated mutant (*, adjusted *P* value = 0.03; ***, adjusted *P* value = 0.0009; and ****, adjusted *P* value < 0.0001).

As a complementary analysis, we performed time-dependent killing tests using 16 μg/mL of Gm (2× MIC) on strains growing anaerobically in LB supplemented or not with NO_3_^–^ or fumarate. Under these conditions, the susceptibility level of the WT strain varied with the nature of the added electron acceptor (Fig. 1A). Indeed, E. coli survived Gm better in the absence of the added electron acceptor (in LB or LB supplemented with glycerol) than in the presence of fumarate or NO_3_^–^ (Fig. 1A). Interestingly, higher survival was observed for cells grown with fumarate compared with NO_3_^–^ (Fig. 1A). To verify that Gm sensitivity was caused by respiratory activity, we tested survival of strains deficient in respiratory chains of NO_3_^–^ or fumarate. NarGHI is the major nitrate reductase, while fumarate respiration is catalyzed by the multicomponent Frd fumarate reductase (FrdABCD). The MIC value of Gm was equivalent to that of the WT (8 μg/mL) for Δ*narG* and Δ*frdA* mutants under either NO_3_^–^ or fumarate respiration, respectively. However, the time-dependent killing experiment showed that the Δ*narG* and Δ*frdA* mutants exhibited an increased level of tolerance to Gm compared to the WT strain (Fig. 1B and C). Overall, these results showed that the respiratory chains have a direct impact on the level of Gm susceptibility and suggest that the lower the energetic yield of the respiratory chain used, the higher the level of Gm survival.

### Hydrogenase-2 and 3, formate dehydrogenase, and MK sensitize E. coli to Gm under fumarate respiration.

**FIG 2:**
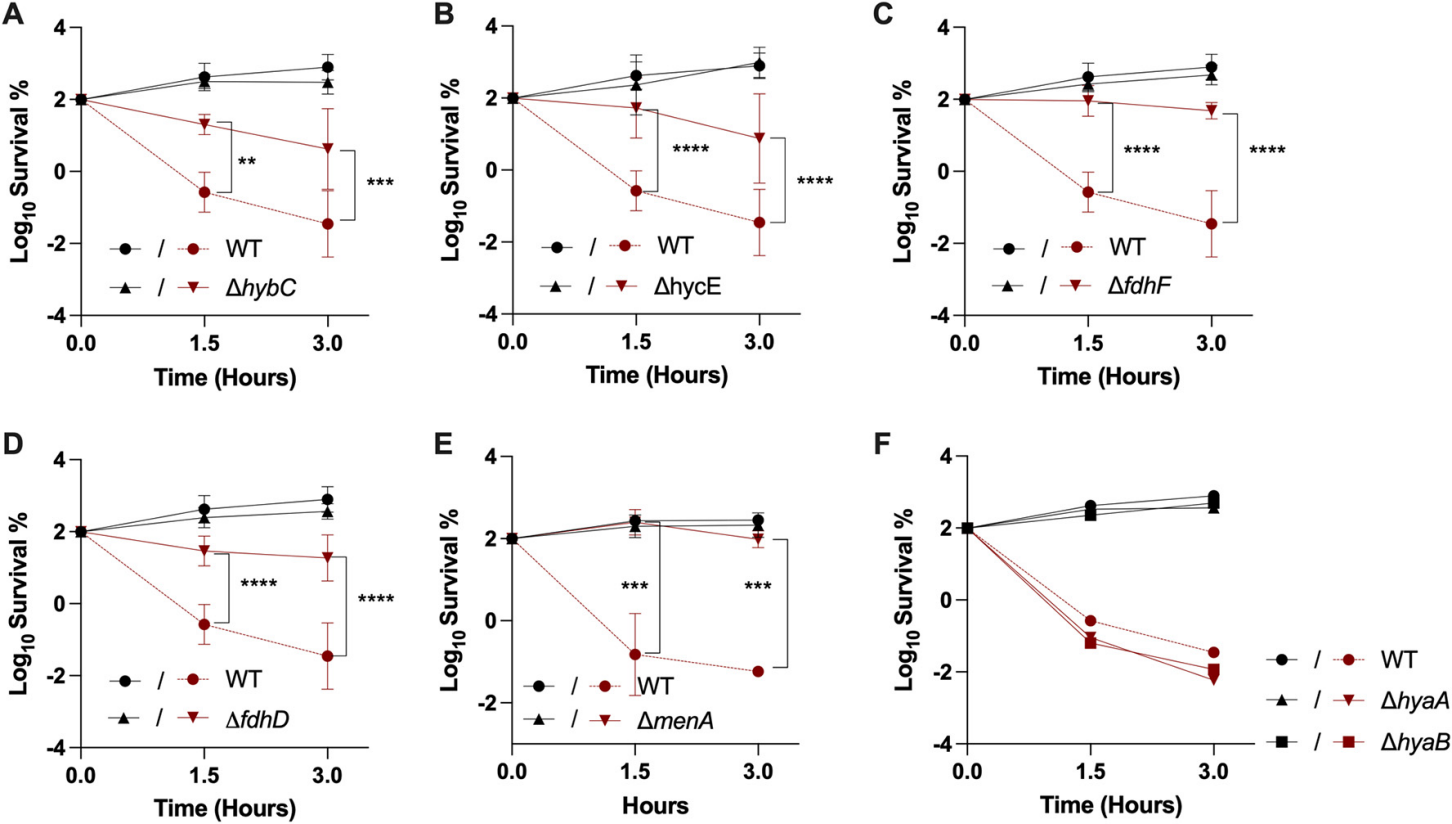
Hydrogenase-2 and 3, formate dehydrogenase, and menaquinone sensitize E. coli to Gm under fumarate respiration. Survival of WT (FBE051), Δ*hybC* (FBE1102) (A), Δ*hycE* (FBE1153) (B), Δ*fdhF* (FBE1157) (C), Δ*fdhD* (FBE1069) (D), Δ*menA* (FBE501) (E), Δ*hyaA* (FBE1117), and Δ*hyaB* (FBE1119) (F) strains after Gm treatment. Cells were grown anaerobically in LB supplemented with fumarate at 10 mM and glycerol at 0.2% and then Gm was added at 16 μg/mL. The survival values after 1.5 and 3 h of treatment are represented. Black and red lines are for untreated and Gm-treated bacteria, respectively. Survival, measured by CFU per mL, was normalized relative to time zero at which Gm was added (early log phase cells; ~5 × 10^7^ CFU/mL) and plotted as Log_10_ of survival percentage. Values are expressed as means of at least three biological replicates and error bars depict standard deviation, except for panel F, which represents the average of two biological replicates. One-way ANOVA tests followed by Sidak’s multiple-comparison tests were performed to compare at each time point (1.5 and 3 h) the treated WT to each of the treated mutant (**, adjusted *P* value = 0.0021; ***, adjusted *P* value = 0.0005; and ****, adjusted *P* value < 0.0001).

We then focused on the low energy-conserving respiratory chain established under fumarate respiration to pinpoint which components are required for Gm sensitization. Fumarate reduction can be coupled to hydrogen oxidation in an electron transfer chain, including Hyd-2, menaquinol-fumarate oxidoreductase FrdABCD as a terminal reductase, and MK and/or DMK (27) as electron carriers (28). To test the contribution of Hyd-2 and/or MK/DMK, we used the Δ*hybC* mutant, lacking the large subunit of Hyd-2, which has primarily hydrogen-uptake activity, and the Δ*menA* mutant, lacking a 1,4-dihydroxy-2-naphthoate octaprenyltransferase required for MK/DMK biosynthesis (29). Time-dependent killing assays were performed in LB medium supplemented with 10 mM fumarate and 0.2% glycerol and using Gm at 16 μg/mL. The Δ*hybC* and Δ*menA* mutants showed increased tolerance to Gm compared to the WT strain (Fig. 2A and E). These results indicated that under fumarate respiration, sensitivity to Gm requires functional Hyd-2 and MK/DMK. Hyd-2 also oxidizes H_2_ produced by the FHL complex. The FHL complex includes formate dehydrogenase (Fdh-H), which produces 2H^+^, 2e^−^, and CO_2_ from formate, and hydrogenase 3 (Hyd-3), which generates molecular H_2_ from 2H^+^ and 2e^−^. We therefore tested the contribution of the FHL complex to the sensitivity to Gm under fumarate respiratory conditions. Specifically, we tested the influence of mutations in genes that affected synthesis of the large subunits of Hyd-3 (*hycE*), formate dehydrogenase H (*fdhF*) and FdhD, an auxiliary maturing factor. Time-dependent killing assays were performed in LB medium supplemented with 10 mM fumarate and 0.2% glycerol and using Gm at 16 μg/mL. The three mutants Δ*hycE*, Δ*fdhF*, and Δ*fdhD* showed increased tolerance of Gm compared with the WT strain (Fig. 2B to D).

E. coli contains another respiratory hydrogenase, Hyd-1, which catalyzes the oxidation of H_2_ to protons and electrons, but does not couple H_2_ oxidation to fumarate reduction (30, 31). We tested the effect of the Hyd-1 complex on Gm susceptibility by exposing Δ*hyaA* or Δ*hyaB* mutants, each lacking a subunit of Hyd-1, to 16 μg/mL Gm. Both mutants exhibited similar antibiotic susceptibility to WT, showing that Hyd-1 does not influence Gm tolerance under such conditions (Fig. 2F).

Fumarate respiration could also involve Nuo or GlpABC complexes as primary electron donors (19). Accordingly, we tested the effect of mutations in genes encoding these two complexes, e.g., *nuoC* and *glpA*, on Gm-mediated killing. Neither the Δ*nuoC* nor the Δ*glpA* mutation, alone or in combination, altered the level of Gm sensitivity (see Fig. S1 in the supplemental material).

10.1128/mbio.03302-22.1 FIG S1 The Nuo complex and the GlpA complex are dispensable for the RavA/ViaA-dependent sensitization of E. coli to Gm under fumarate respiration. (A, B, C) Survival of WT (FBE051), Δ*nuoC* (FBE1057), Δ*glpA* (FBE950), and Δ*glpA* Δ*nuoC* (FBE1055) strains after Gm treatment. Cells were grown in LB supplemented with fumarate at 10 mM (A) and glycerol at 0.2% (B, C) and then Gm was added at 16 μg/mL. The survival values after 1.5 and 3 h of treatment are represented. Black and red lines are for untreated and Gm-treated bacteria, respectively. The lines of untreated cells are overlapping. Survival measured by CFU per mL, was normalized relative to time zero at which Gm was added (early log phase cells; ~5 × 10^7^ CFU/mL) and plotted as Log_10_ of % survival. For (A) and (B), values are expressed as means of at least 3 biological replicates and error depict standard deviation. One-way ANOVA tests followed by Sidak’s multiple comparison tests were performed to compare at each time point (1.5 and 3 h) the treated WT to each of the treated mutant (ns = not significant). Download FIG S1, DOCX file, 0.2 MB. Copyright © 2023 El Khoury et al. 2023 El Khoury et al. https://creativecommons.org/licenses/by/4.0/ This content is distributed under the terms of the Creative Commons Attribution 4.0 International license.

Taken together, these results demonstrate that in E. coli growing anaerobically in the presence of fumarate (and glycerol), Gm sensitivity is caused by the activity of the FHL-Hyd-2-MK/DMK-FrdA electron transfer chain.

### RavA-ViaA sensitizes E. coli to Gm under anaerobic fumarate respiration conditions.

**FIG 3:**
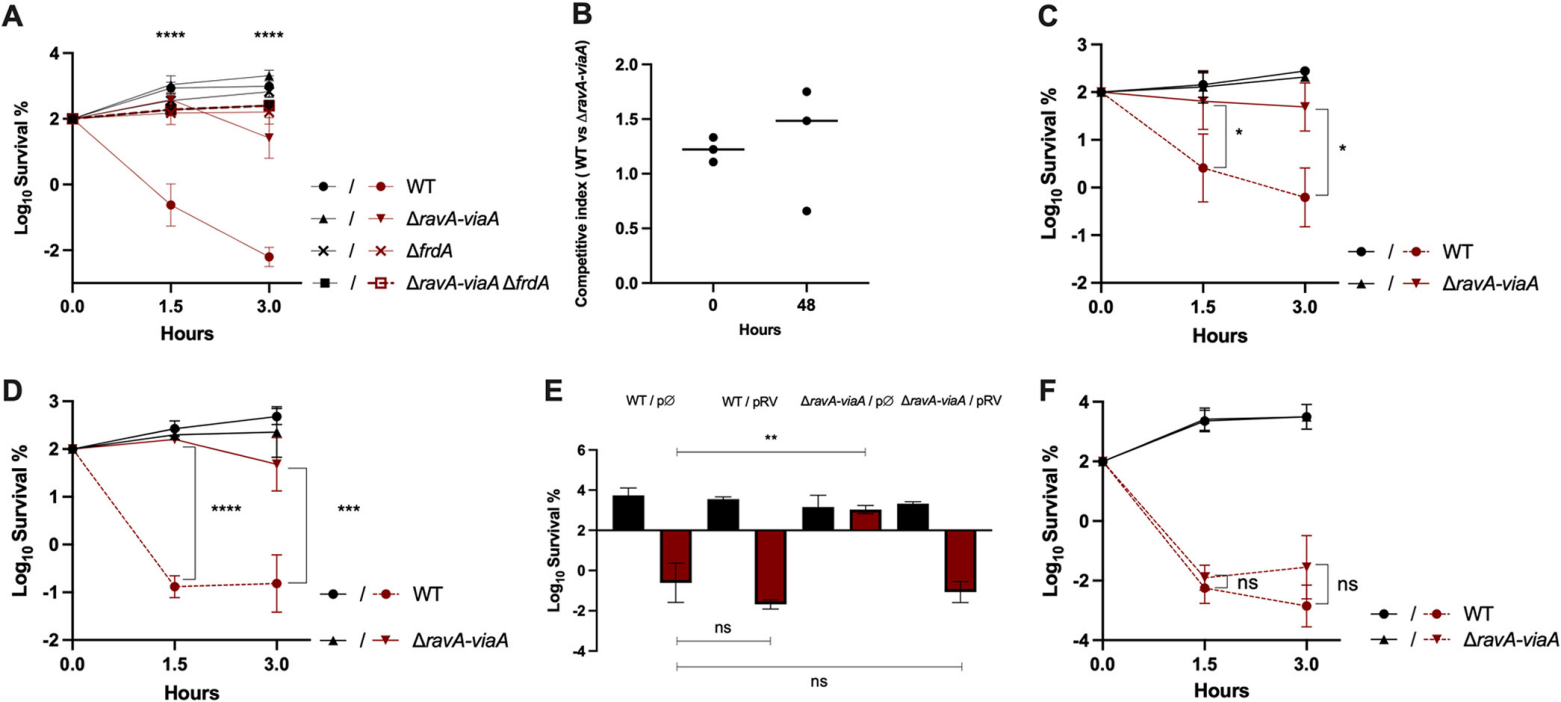
RavA-ViaA sensitizes E. coli to Gm under specific anaerobic conditions. (A and B) RavA-ViaA (RV) sensitizes E. coli to Gm under anaerobic fumarate respiratory conditions. (A) Survival of WT (FBE051), Δ*ravA-viaA* (FBE706), Δ*frdA* (FBE790), and Δ*ravA-viaA* Δ*frdA* (FBE831) strains after Gm treatment. Cells were grown anaerobically in LB supplemented with fumarate at 10 mM and then Gm was added at 16 μg/mL. For the Δ*ravA-viaA* Δ*frdA* (FBE831) strain, lines of treated and untreated cells are overlapping. (B) WT (FBE051) and Δ*ravA-viaA*::CmR (BP970) were grown separately overnight in M9 minimal medium supplemented with fumarate (10 mM), glycerol (0.2%), and casamino acids (0.1%). Cultures were then diluted 1/100 into fresh medium and coinoculated in a 1:1 ratio (t0). The coculture was incubated for 48 h at 37°C for a competitive growth. The competitive index was calculated as follows: (CFU_mutant_/CFU_WT_)_t48_/(CFU_mutant_/CFU_WT_)_t0_. Lines represent the median values of three independent experiments. (C to E) RavA-ViaA sensitizes E. coli to Gm in the absence of an exogenously added electron acceptor in anaerobiosis: survival of WT (FBE051) and the Δ*ravA-viaA* (FBE706) strains after Gm treatment. Cells were grown in LB (C) or in LB supplemented with 0.2% glycerol (D) until OD_600_ reached ~0.2 and then Gm was added at 16 μg/mL. (E) Survival of WT (FBE051) and the Δ*ravA-viaA* (FBE706) strains carrying either the pRV plasmid or the control empty vector (pØ). Cells were grown in LB supplemented with glucose (0.2%), IPTG (1 mM), and ampicillin (50 μg/mL) until OD_600nm_ reached ~0.2 and then Gm was added at 30 μg/mL. The survival values after 3 h of treatment are represented. Black and red bars are for untreated and antibiotic-treated bacteria, respectively. Survival, measured by CFU per mL, was normalized relative to time zero at which Gm was added (early log phase cells; ~5 × 10^7^ CFU/mL) and plotted as Log_10_ of survival percentage. One-way ANOVA tests followed by Dunnett’s multiple-comparison tests were performed to compare the treated WT to the treated Δ*ravA-viaA* mutant (ns, not significant and **, adjusted *P* value < 0.05). (F) RavA-ViaA does not sensitize E. coli grown under nitrate respiration to Gm: survival of WT and Δ*ravA-viaA* strains after Gm treatment. Cells were grown anaerobically in LB supplemented with NO_3_^–^ at 10 mM and glycerol at 0.2% and then Gm was added at 16 μg/mL. Lines of untreated WT and Δ*ravA-viaA* (FBE706) strains are overlapping. (A, C, D, and F) The survival values after 1.5 and 3 h of treatment are represented. Black and red lines are for untreated and Gm-treated bacteria, respectively. Values are expressed as means of at least three biological replicates and error bars depict standard deviation. One-way ANOVA tests followed by Sidak’s multiple-comparison tests were performed to compare at each time point (1.5 and 3 h) the treated WT to the treated mutants (*, adjusted *P* value < 0.05; ***, adjusted *P* value = 0.0002; and ****, adjusted *P* value < 0.0001).

It has been proposed that the RavA-ViaA complex acts as a chaperone by assisting in the assembly of fumarate reductase (22). Therefore, we tested the effect of the RavA-ViaA complex on the AG sensitivity of E. coli grown under anaerobic fumarate respiration conditions. The Δ*ravA-viaA* mutant exhibited the MIC value of Gm during fumarate respiration equivalent to that for the WT (8 μg/mL). However, a time-dependent killing experiment using a Gm concentration equivalent to 2× MIC (16 μg/mL) revealed a significant increase in Gm tolerance of the mutant compared to the WT strain (Fig. 3A). The combination of Δ*ravA-viaA* and Δ*frdA* mutations showed no additive effect (Fig. 3A).

A previous analysis showed that RavA-ViaA exerted a slight negative effect on FrdA enzymatic activity (22). We therefore tested whether RavA-ViaA might cause an energetic disadvantage in strains growing using fumarate respiration. WT and Δ*ravA*-*viaA* strains were mixed in a 1:1 cellular ratio and grown together in M9-glycerol and fumarate under anaerobic conditions for 48 h. The competitive index was determined by counting CFU of the Δ*ravA*-*viaA* strain, using its chloramphenicol-resistance phenotype, and CFU of the WT at t_0_ and t_48_. The competitive index (CFU_mutant_/CFU_wt_)t_48_/(CFU_mutant_/CFU_wt_)t_0_ yielded a median value of 1.3, revealing no growth advantage of one strain over the other (Fig. 3B). These results showed that RavA-ViaA *in vivo* does not influence growth under fumarate respiration and presumably does not modulate FrdA enzymatic activity to a significant extent, if at all.

Taken together, these results indicate that under fumarate respiration conditions, the RavA-ViaA complex sensitizes E. coli to Gm via a FrdA-dependent mechanism.

### RavA-ViaA sensitizes E. coli to Gm in the absence of an exogenously added electron acceptor under anaerobic conditions.

Next, we tested whether fumarate was required for the RavA-ViaA complex to exert its sensitizing effect. Thus, E. coli was grown in the absence of an exogenously added electron acceptor, i.e., in LB medium, LB-0.2% glycerol or LB-0.2% glucose. First, MIC values for Gm in all these media were found to be 8 μg/mL. Next, we performed a killing assay using Gm at 16 μg/mL (2× MIC) in LB (Fig. 3C) and in LB glycerol (Fig. 3D), and at 30 μg/mL (~4× MIC) in LB glucose (Fig. 3E). The Δ*ravA-viaA* mutant showed increased tolerance to Gm compared to the WT strain in all media. Notably, complementation of the Δ*ravA-viaA* mutant with a multicopy plasmid (pRV plasmid) containing the *tac_p_*::*ravA-viaA* operon allele, suppressed its increased tolerance (Fig. 3E). These results indicated that RavA-ViaA exerts a sensitizing effect on E. coli under anaerobic conditions even in the absence of an added exogenous electron acceptor.

### RavA-ViaA does not sensitize E. coli grown under nitrate respiration to Gm.

Next, we tested whether RavA-ViaA was able to sensitize E. coli to Gm under NO_3_^–^ respiration. First, we determined the MIC value of Gm in LB medium supplemented with 10 mM NaNO_3_ and 0.2% glycerol and found it to be 8 μg/mL for both WT and Δ*ravA-viaA* strains. Under the same conditions, a time-dependent killing assay done with Gm at 16 μg/mL showed that the Δ*ravA-viaA* mutant was as susceptible as the WT strain (Fig. 3F). These results indicated that the sensitization of E. coli to Gm under NO_3_^–^ respiration is independent of RavA-ViaA.

### RavA-ViaA sensitizes E. coli to AG specifically.

**FIG 4:**
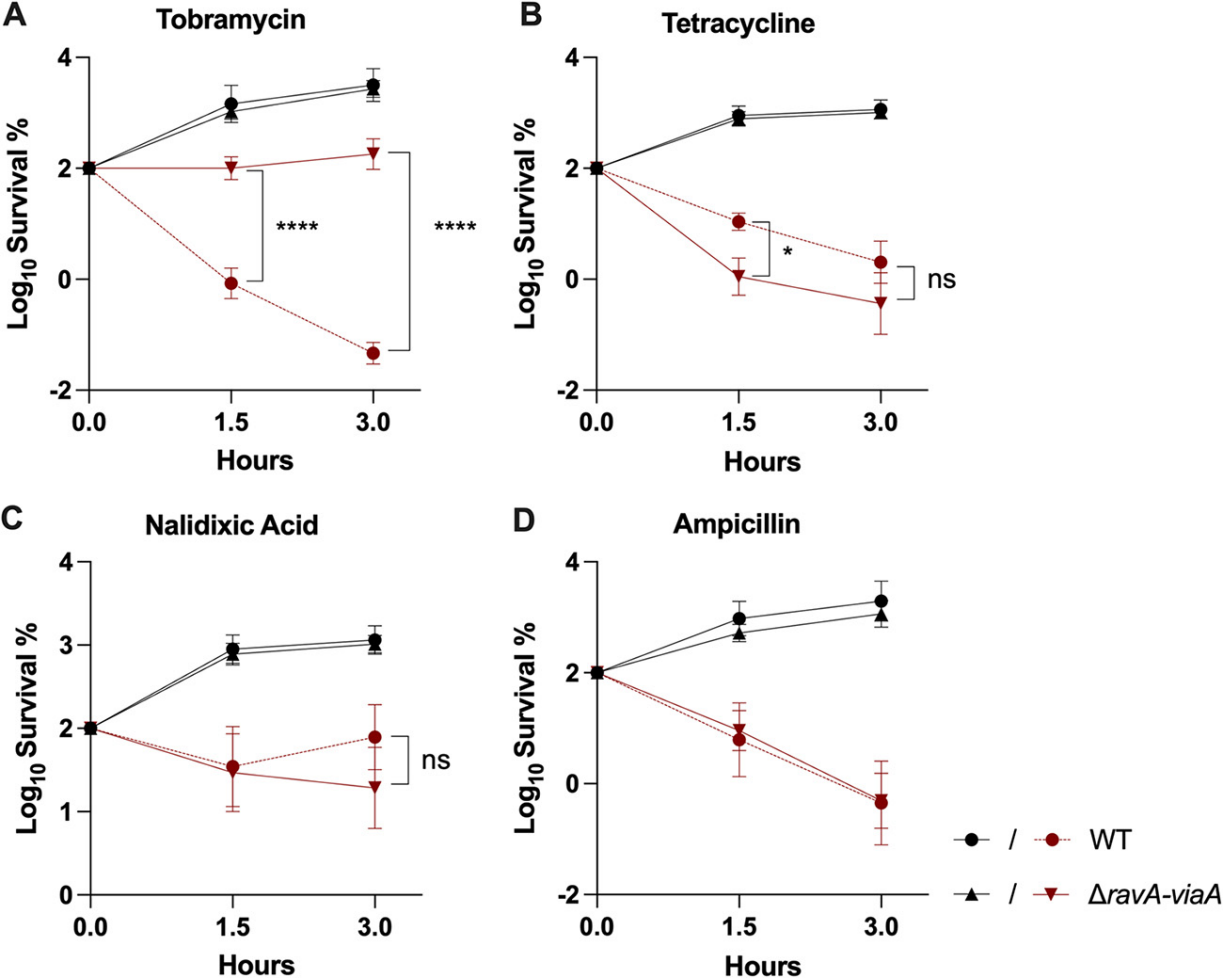
RavA-ViaA sensitizes E. coli specifically to AG. Survival of WT (FBE051) and Δr*avA-viaA* (FBE706) strains after antibiotic treatment. Cells were grown in LB supplemented with glucose (0.2%) until OD_600_ reached ~0.1 and antibiotics were added: (A) tobramycin (30 μg/mL); (B) tetracycline (5 μg/mL); (C) nalidixic acid (5 μg/mL); and (D) ampicillin (5 μg/mL). Black and red lines are for untreated and antibiotic-treated bacteria, respectively. The survival values after 1.5 and 3 h of treatment are represented. Survival, measured by CFU per mL, was normalized relative to time zero at which the antibiotic was added and plotted as Log_10_ of survival percentage. Values are expressed as means (*n* = 3) and error bars depict standard deviation. One-way ANOVA tests followed by Sidak’s multiple-comparison tests were performed to compare at each time point (1.5 and 3 h) the treated WT to the treated Δ*ravA-viaA* mutant (ns, not significant; *, adjusted *P* value < 0.05; and ****, adjusted *P* value < 0.0001).

To investigate the spectrum of the sensitizing effect of the RavA-ViaA complex, we measured the survival rate of the Δ*ravA*-*viaA* strain to another AG (tobramycin), a protein synthesis inhibitor (tetracycline), a fluoroquinolone (nalidixic acid), and a β-lactam (ampicillin). We observed that the Δ*ravA-viaA* mutant was more tolerant to tobramycin than the WT strain (Fig. 4A). A modest tolerance effect was noted upon short time exposure to tetracycline (Fig. 4B). The Δ*ravA*-*viaA* mutation had no effect on the potency of nalidixic acid and ampicillin (Fig. 4C and D). Overall, these results showed that under anaerobic conditions, RavA-ViaA sensitizes E. coli specifically to AG.

### RavA-ViaA increases intracellular Gm concentration under anaerobic conditions.

**FIG 5:**
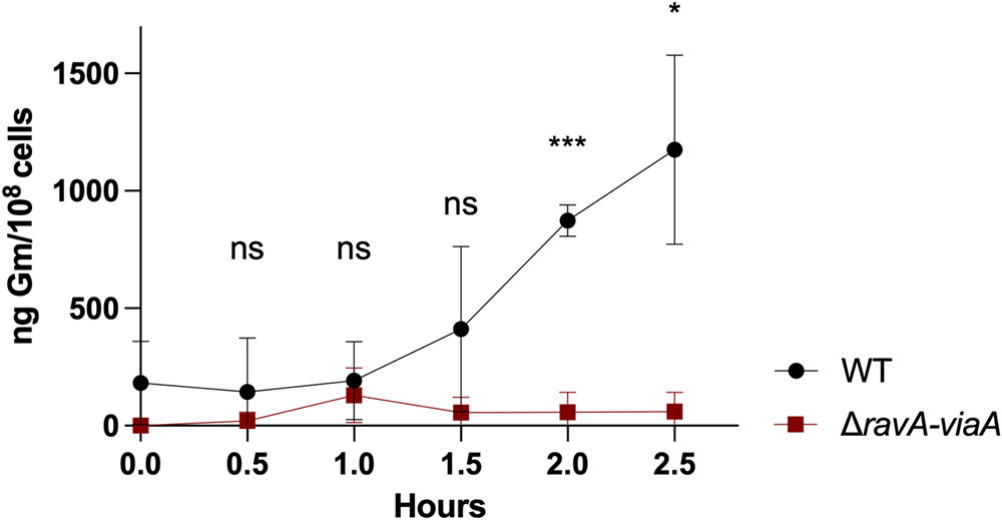
RavA-ViaA increases Gm uptake. ^3^H-Gm uptake in WT (FBE051) and Δ*ravA-viaA* (FBE706) strains was measured by incubating early exponential-phase cultures (OD_600_ = ~0.1) with 30 μg/mL ^3^H-Gm at 37°C under anaerobic conditions (LB supplemented with glucose at 0.2%). Values are expressed as means (*n* = 3) and error bars depict standard deviation. Unpaired *t* test followed by Welch’s correction was performed to compare the WT strain to the Δ*ravA-viaA* mutant at each time point (ns, not significant; *, adjusted *P* value < 0.05; and ***, adjusted *P* value = 0.0003).

To understand the role of RavA-ViaA in AG sensitization under anaerobic conditions, we performed a Gm uptake assay using ^3^H-Gm. The accumulation of ^3^H-Gm in the WT strain gradually increased to 1,200 ng Gm/10^8^ cells after 2.5 h (Fig. 5). In contrast, in the Δ*ravA-viaA* mutant, ^3^H-Gm accumulation remained below 100 ng of Gm/10^8^ cells after 2.5 h. Our results indicated that RavA-ViaA sensitizes E. coli to Gm by increasing its uptake and, consequently, its intracellular concentration.

### Increased dosage of the *ravA-viaA* gene enhances AG-mediated killing of E. coli in aerobiosis.

**FIG 6:**
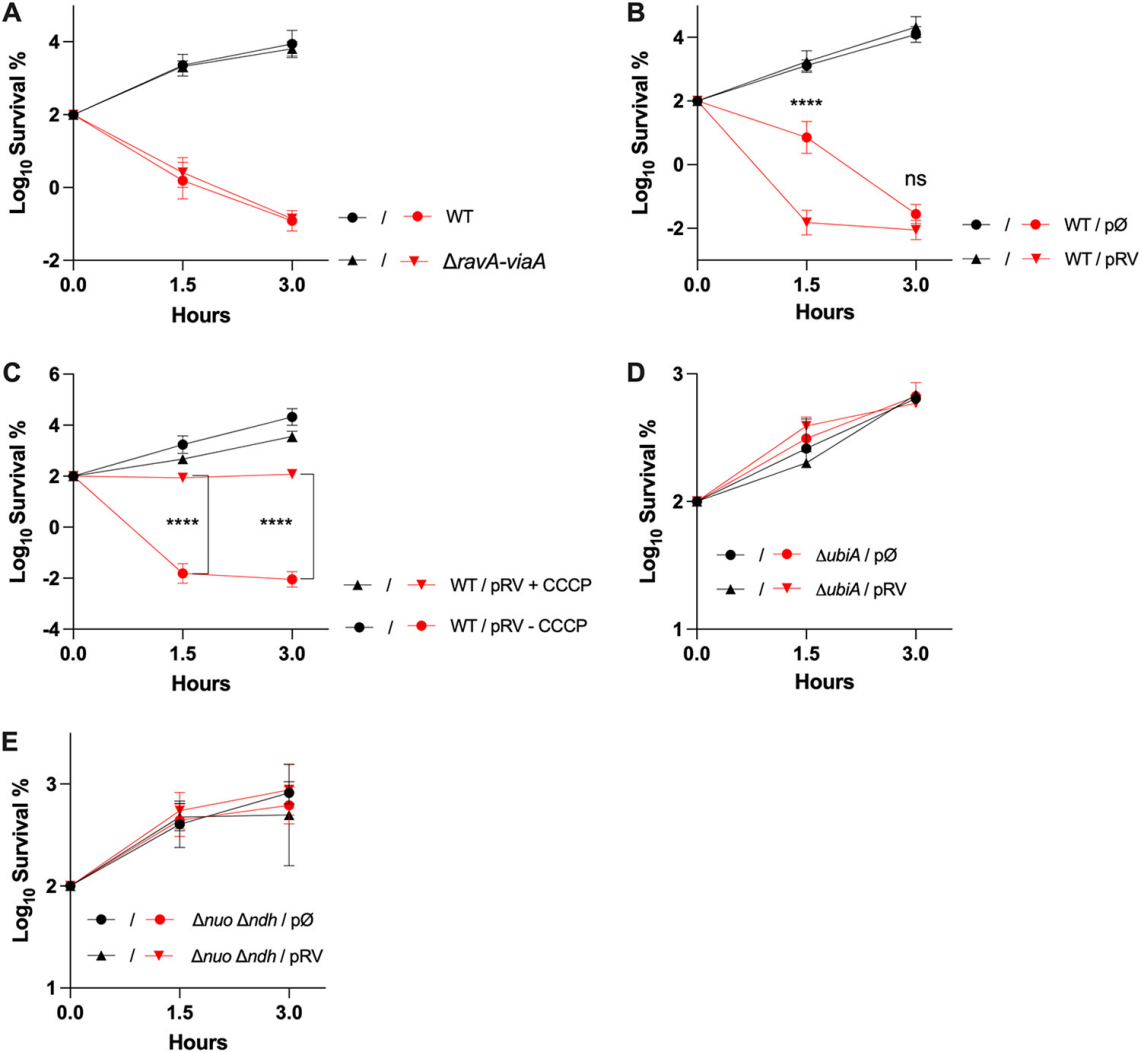
Effect of RavA-ViaA on E. coli sensitivity to Gm under aerobiosis. (A and B) Increased *ravA* and *viaA* genes dosage alters survival to Gm in killing assay. Survival of the strain WT (FBE051) and the mutant Δ*ravA-viaA* (FBE706) (A) or of the WT strain containing either a plasmid that carries the *ravA-viaA* operon (pRV) or the empty vector control (pØ) (B) after treatment with Gm (5 μg/mL) for 1.5 and 3 h. (C to E) The RavA-ViaA Gm sensitization phenotype is abolished by *pmf* inhibitor and is dependent upon a functional respiratory chain. (C) Survival of the WT strain (FBE051) containing a plasmid that carries the *ravA-viaA* operon (pRV) after treatment with Gm (5 μg/mL), in the presence or absence of CCCP (5 μg/mL). Survival of Δ*ubiA* (LL922) (D) and Δ*nuo* Δ*ndh* (BP1046) (E), containing a plasmid that carries the *ravA-viaA* operon (pRV) or the empty vector control (pØ), after Gm treatment. Cells were grown in LB supplemented with IPTG (1 mM) and ampicillin (50 μg/mL) until OD_600_ reached ~0.1 and Gm (5 μg/mL) was added. (A to E) Survival, measured by CFU per mL, was normalized relative to time zero, at which the antibiotic was added (early log phase cells; ~5 × 10^7^ CFU/mL) and plotted as Log_10_ of survival percentage. Values are expressed as means (*n* = 3) and error bars depict standard deviation. Black and red lines are for untreated and Gm-treated, respectively. One-way ANOVA tests followed by Sidak’s multiple-comparison tests were performed (ns, not significant and ****, adjusted *P* value < 0.0001).

We decided to investigate whether the RavA-ViaA complex could have a sensitizing effect under aerobic conditions. The MIC of Gm was found to be 2 μg/mL for both wild type (WT) and Δ*ravA-viaA* strains. In a time-dependent killing experiment using a concentration of Gm equivalent to 2.5× MIC (5 μg/mL), E. coli WT and Δ*ravA-viaA* strains exhibited similar sensitivities to Gm (Fig. 6A). However, because the expression of the *ravA-viaA* operon is under Fnr control (22), we reasoned that the genes might not be expressed at a high enough level under the conditions used. Therefore, we bypassed Fnr-mediated activation using the pRV plasmid, and tested whether this could sensitize E. coli to Gm. We observed significantly impaired survival of the WT/pRV strain (Fig. 6B), demonstrating the ability of RavA-ViaA to sensitize E. coli to Gm-mediated killing under aerobiosis. Thus, we concluded that the RavA-ViaA complex can also sensitize E. coli to Gm under aerobic respiration, but only when they are produced above a threshold value, a situation that does not appear to be achieved in exponentially growing cells harboring a chromosomal copy of the *ravA-viaA* operon.

### RavA-ViaA-mediated killing of E. coli by AG in aerobiosis requires *pmf*.

Survival of the WT/pRV strain exposed to Gm was tested in the presence of cyanide-m-chlorophenylhydrazone (CCCP), an ionophore, which dissipates *pmf*. The data showed that addition of CCCP prevented Gm from killing the WT/pRV strain (Fig. 6C), demonstrating that pRV-mediated killing required *pmf*. Because *pmf* results from respiratory metabolism, we tested whether pRV-mediated sensitization was dependent on electron transfer chain (ETC)-forming components. We analyzed the survival of strains defective for the synthesis of ubiquinones, the lipid that acts as an electron transporter in aerobic ETCs. A Δ*ubiA* strain was highly resistant to Gm treatment and the pRV plasmid failed to sensitize this strain to Gm (Fig. 6D). Similarly, a Δ*nuo* Δ*ndh* mutation altering NADH dehydrogenase I and II abrogated pRV-mediated sensitization (Fig. 6E). These data supported the idea that respiration is required for pRV-mediated killing of E. coli by Gm in aerobiosis.

## DISCUSSION

AGs have been used for decades to treat Gram-negative infections. In this work, we report how the type of respiratory metabolism used by E. coli can influence the level of sensitivity to AG. We identify the molecular components of the respiratory chain and an AAA+ ATPase RavA, associated with its VWA-containing partner, ViaA, needed for AG to act in low energy-producing fumarate respiration. We propose that the level of sensitivity increases inversely with the energy yield of the respiratory chain used and that the contribution of the RavA-ViaA complex occurs when cells are in a low energy state.

**FIG 7:**
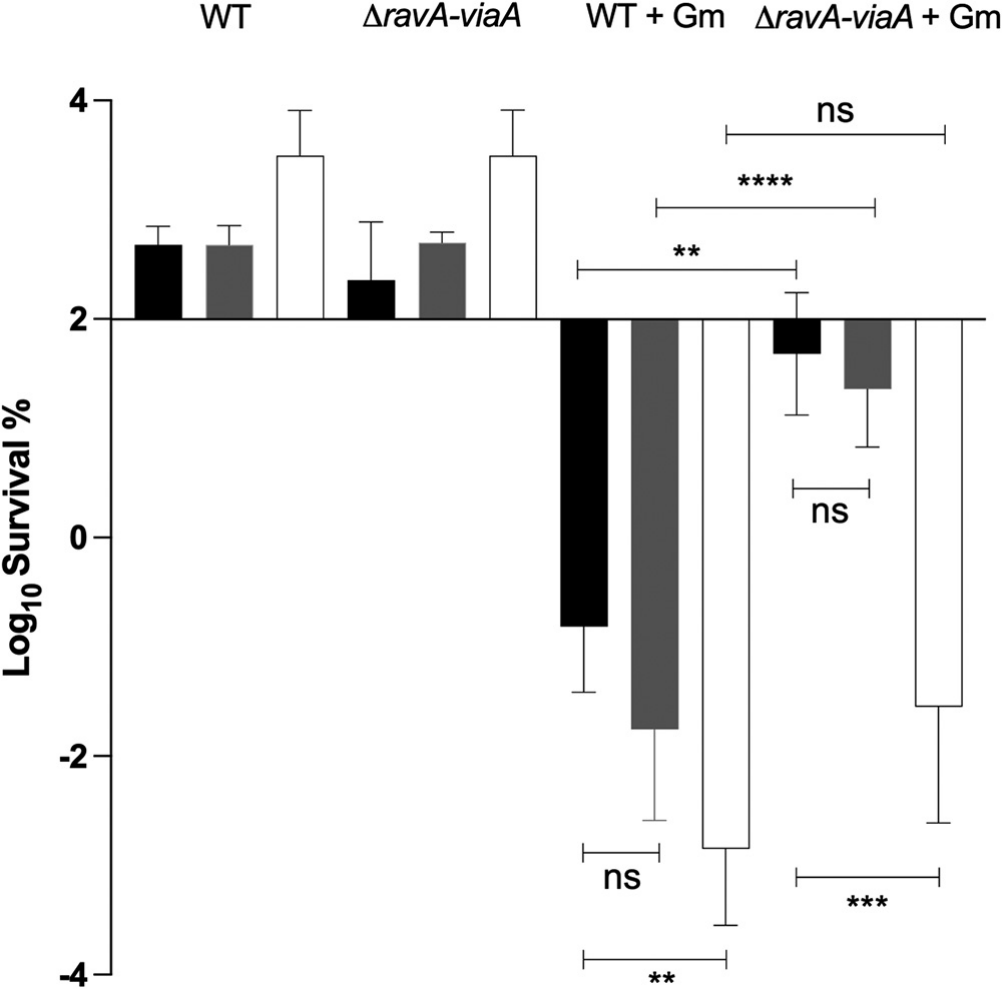
Energy conservation level affects Gm sensitivity under anaerobic conditions. Survival of WT (FBE051) and the Δ*ravA-viaA* (FBE706) strains after Gm treatment. Cells were grown in LB-glycerol (black), supplemented with 10 mM fumarate (gray) or with 10 mM nitrate (white) until OD_600_ reached ~0.2 and then Gm was added at 16 μg/mL. The survival values after 3 h of treatment are represented. The first two groups represent the untreated strains, and the two last groups represent the treated strains (+Gm). Values are expressed as means of at least three biological replicates and error bars depict standard deviation. One-way ANOVA tests followed by Sidak’s multiple-comparison tests were performed (ns, not significant; *, adjusted *P* value < 0.05; **, adjusted *P* value < 0.005; ***, adjusted *P* value = 0.0003; and ****, adjusted *P* value < 0.0001).

E. coli has a highly versatile arsenal of respiratory chains. E. coli synthesizes multiple dehydrogenases and terminal reductases, which act as quinone reductases and oxidases, respectively (20, 32). Thus, from a bioenergetic standpoint, quinones can link most dehydrogenases to most reductases and a wide variety of respiratory chains can be formed (20, 32). Energy conservation is maximal in aerobiosis, decreases under fumarate respiration and reaches its lowest level in the absence of an exogenously added electron acceptor (20). Accordingly, we found that the predicted level of energy conservation parallels the level of Gm sensitivity (Fig. 1 and 7).

Components of the respiratory chain enabling AG sensitivity under fumarate respiration include FHL, Hyd-2, MK, and Frd. Regarding nitrate respiration, similar systematic dissection of the components required for AG sensitivity will be of interest, particularly the role of the recently discovered anaerobic ubiquinone biosynthesis pathway, as nitrate reductase uses both ubiquinol and menaquinol as electron donor (33). We also identified the RavA-ViaA complex as being required to mediate AG toxicity under such conditions. RavA belongs to the AAA+ ATPase MoxR family, whose physiological role remains to be elucidated (34). We have shown that the RavA-ViaA complex sensitizes E. coli to AG through a *pmf*-dependent mechanism. The simplest explanation for the sensitizing role of RavA-ViaA would be that it increases respiratory chain activity, resulting in elevated *pmf* level and enhanced Gm uptake. Yet, this explanation seems to be insufficient, because no positive effect of RavA-ViaA complex was found either on Frd or on Nuo activity (Fig. S2) and no advantage was associated with RavA-ViaA complex for growth on fumarate. Recently, we reported that RavA-ViaA localizes to the inner membrane where it interacts with lipids and the lipid-binding properties of the RavA-ViaA complex were found to be necessary for AG sensitization under fumarate respiration (35). Thus, it seems a reasonable hypothesis to propose that the RavA-ViaA complex acts on the membrane to optimize function of some of the respiratory chains synthesized by E. coli, such as that involving Frd.

10.1128/mbio.03302-22.2 FIG S2 RavA-ViaA have no effect on Nuo activity. Nuo specific activity in the WT (FBE051) and the Δ*ravA-viaA* (FBE706) strains containing the plasmid carrying the *ravA-viaA* genes (pRV) or the corresponding empty vector (pØ). Nuo specific activity was measured in cells extracts using deamino-NADH as the substrate. Values are expressed as means (*n* ≥ 3) and error bars depict mean deviation. One-way ANOVA tests followed by Dunnett’s multiple comparison tests were performed (ns = not significant). The 100% corresponding to the activity in the WT strain is 127 nmol/min/mg protein. Download FIG S2, DOCX file, 0.1 MB. Copyright © 2023 El Khoury et al. 2023 El Khoury et al. https://creativecommons.org/licenses/by/4.0/ This content is distributed under the terms of the Creative Commons Attribution 4.0 International license.

Surprisingly, the extent of RavA-ViaA contribution to E. coli sensitization to Gm appears to follow the redox energy hierarchy. Indeed, the influence of RavA-ViaA was apparent with the exogenous electron acceptors providing the lowest energy conservation, i.e., without addition of an exogenous electron acceptor or with fumarate (Fig. 7). It is not clear which mode E. coli is relying on to grow in the absence of an “added exogeneous electron acceptor” and we assume that trace levels of NO_3_^–^ and/or amino acids present in the rich medium acted as electron acceptors. In contrast, RavA-ViaA had no significant influence when the electron acceptors were NO_3_^–^ or O_2_, which both give rise to the two highest energy conservation processes. Thus, the sensitizing effect of RavA-ViaA occurs only when cellular energy diminishes under a threshold value. Although this does not provide a clue as to its molecular mechanism, this points to a role of stress sensing, which fits in the chaperone role of AAA+ ATPases.

The *ravA-viaA* operon is under the control of Fnr (22) and we consistently found a phenotype for the Δ*ravA-viaA* mutant in anaerobiosis. In contrast, killing assays failed to show a change in the level of Gm tolerance of the *ΔravA-viaA* mutant in the presence of O_2_. Yet, increased dosage of the *ravA-viaA* gene sensitized E. coli to Gm, indicating that aerobic conditions were not inherently inhibitory to RavA-ViaA activity. The previously reported involvement of RavA-ViaA in sensitivity to AG in O_2_ was derived from examination of the final optical density at 600 nm (OD_600_) value reached by E. coli grown in the presence of sublethal concentrations of kanamycin, another AG. Our phenotypic analysis of the Δ*ravA-viaA* mutant did not confirm a role for *ravA-viaA* in AG tolerance in the presence of O_2_. However, our protocol relied on killing assays with strains taken in the mid-exponential phase. Because, the *ravA-viaA* operon is under the control of sigma S (22), it is likely that in our experiments, RavA and ViaA protein levels were not high enough for an influence to be detected. It will be interesting to revisit the question of the influence of RavA-ViaA on Gm lethal activity on stationary resting cells. We note that the model of an increased effect of RavA-ViaA on Gm tolerance of cells in stationary phase somehow meets with the hypothesis stated above of an effect of RavA-ViaA on cells under low energy/stress conditions. Moreover, the effect of RavA-ViaA on AG susceptibility was also observed under fermentative conditions, i.e., in LB-glucose. Altogether these observations support a role of RavA-ViaA in *pmf* sensing rather than respiratory activity *per se*. Next studies will aim to identify how the RavA-ViaA complex might harness, or influence, *pmf* to enhance AG uptake.

Antimicrobials often fail to cure the infection and, in addition to resistance, some of the reasons lie in metabolism used by bacteria to adapt to environmental conditions. Here, we have described how environmental conditions and the presence/absence of compounds with a potential electron acceptor role can alter the level of tolerance of E. coli to AG. In addition, we identified key molecular players that sensitize E. coli to AG under anaerobiosis. AGs have been used for decades, but their potential toxicity has limited their use. Elucidation of the mechanisms of AG sensitization in bacteria will enable the use of a reduced effective dose of AGs to safely treat a greater proportion of infections. Finally, we wish to highlight how a molecular understanding of the relationship between the type of respiratory electron acceptor available in the environment and bacterial susceptibility to antibiotics could help to understand dynamics of the microbiota. Indeed, it has been proposed that the nature of the respiratory electron acceptor present in the different sections of the intestine (duodenum, illium, and cecum) directly controls the type of bacterial population found there (36). Thus, one could predict the level of tolerance that enterobacteria such as E. coli will show, depending upon its location in the digestive tract.

## MATERIALS AND METHODS

### Bacterial strains and growth conditions.

The E. coli K-12 strain MG1655 and its derivatives used in this study are listed in Table 1. Deletion mutations (Δ*hyaA*::Kan^R^, Δ*hyaB*::Kan^R^, Δ*hybC*::Kan^R^, Δ*hycE*::Kan^R^, Δ*fdhD*::Kan^R^, Δ*fdhF*::Kan^R^, Δ*frdA*::Kan^R^, Δ*glpA*::Kan^R^, Δ*menA*::Kan^R^, Δ*narG*::Kan^R^, Δ*ndh*::Kan^R^, Δ*nuoC*::Kan^R^) from the KEIO collection were introduced by P1 transduction. The Δ*ravA-viaA*::Cm^R^ mutant was constructed using the procedure described by Datsenko K. and Wanner B. (37), using oligonucleotides ravAwanner_up and viaAwanner_do (Table 2), followed by a transduction in MG1655 background. Transductants were verified by PCR, using primer pair hybridizing upstream and downstream the deleted genes. When performed, excision of the antibiotic cassette was done using the pCP20 plasmid (37). Oligonucleotides used in this study are listed in Table 2. E. coli strains were grown at 37°C in Luria-Bertani (LB) rich medium or in minimal M9 medium. Glucose (0.2%), glycerol (0.2%), IPTG (1 mM), CCCP (5 μg/mL), fumarate (10 mM), or NO_3_^–^ (10 mM) were added when indicated. Solid media contained 1.5% agar. For standard molecular biology techniques, antibiotics were used at the following concentrations: chloramphenicol at 25 μg/mL, kanamycin at 50 μg/mL, and ampicillin at 50 or 100 μg/mL.

**TABLE 1:** E. coli strains used in this study

E. coli strains	Relevant genotype	Source
BP897	MG1655 Δ*nuo*::nptI Kan^R^	Ezraty B. et al. (8)
BP1046	MG1655 Δ*nuo::nptI* Δ*ndh*::FRT^*a*^	This study
FBE051	MG1655 Wild type	Lab collection
FBE501	MG1655 Δ*menA*::FRT	This study
BP970	MG1655 Δ*ravA-viaA*::Cm^R^	This study
FBE706	MG1655 Δ*ravA-viaA*::FRT	This study
FBE790	MG1655 Δ*frdA*::FRT	This study
FBE829	MG1655 Δ*narG*::FRT	This study
FBE831	MG1655 Δ*ravA-viaA* Δ*frdA*::FRT	This study
FBE950	MG1655 Δ*glpA*::FRT	This study
FBE1055	MG1655 Δ*glpA* Δ*nuoC*::FRT	This study
FBE1057	MG1655 Δ*nuoC*::FRT	This study
FBE1069	MG1655 Δ*fdhD*::FRT	This study
FBE1102	MG1655 Δ*hybC*::FRT	This study
FBE1117	MG1655 Δ*hyaA*::FRT	This study
FBE1119	MG1655 Δ*hyaB*::FRT	This study
FBE1153	MG1655 Δ*hycE*::FRT	This study
FBE1157	MG1655 Δ*fdhF*::FRT	This study
LL922	MG1655 Δ*ubiA*	Kazemzadeh K. et al. (39)

a Elimination of the antibiotic resistance cassette by Flippase (FLP)-promoted recombination events leaves an FRT scar (Flippase recognition target).

**TABLE 2:** Oligonucleotides used in this study

Primer name	Sequence (5′–3′)
Construction of the Δ*ravA*-*viaA* mutant	
ravAwanner_up	CTCGCAATTTACGCAGAACTTTTGACGAAAGGACGCCACTTCATTTGTGTAGGCTGGAGC
viaAwanner_do	GCCAGCTGCTGTTCGCGAGAGCGTCCCTTCTCTGCTGTAATAATCATATGAATATCCTCC
Construction of the pRV plasmid	
ravA UP BamHI	GGCCGGATCCATGGCTCACCCTCATTTATTA
viaA DO HindIII	GGCCAAGCTTTTATCGCCGCCAGCGTCTGAG
Checking the knockout mutants	
fdhD_Frd	TTTCTTTGCGGAAGGGGCCG
fdhD_Rv	GAAAACGCCACTACACGCATTT
fdhF_Frd	CGAATGGATAAAAAAACAGCCTCCG
fdhF_Rv	ATGACCCCACATAAAATGTGGC
frdA_Frd	GTGGAATAGCGTTCGCAGACC
frdA_Rv	GCTATGCGGTGCGGTATCGAC
glpA_Frd	ATGAGCGAATATGCGCGAAATCAAA
glpA_Rv	GCAGTTGCAGGCCACAGAGTAA
hyaA_Frd	ATGGTTTGCCTTGCTACAGGGA
hyaA_Rv	GCGGCGTCCGGCATTATTG
hyaB_Frd	GACCAGCGCAGACGTCATAAC
hyaB_Rv	GTGGCTGACAACGTTGTCGC
hybC_Frd	CCGATGGCTTCATCGGTCAG
hybC_Rv	CGTACTCATTCGTCTACTGCCG
hycE_Frd	ATAAGACGAGGTCGCCGTGC
hycE_Rv	TATTACTCCGCGCATTACCTGGG
menA_Frd	AACATCTGGATGCGTTGGTGG
menA_Rv	TAGGCTTAACATTCAGTTGCTGC
narG_frd	AGGCTCCCACAGGAGAAAACCG
narG_Rv	CACCATGCCGACTTGTGAACGAATTT
nuoC_Frd_2	TGCTCGATCGCTTCACGCTC
nuoC_Rv_2	TCGGCAAAGGGATTTTTCTTCGC
ravA_up_verif	CCTAAATGCGGCCACATTAACC
viaA_do_verif	GGCGGCGGTATCGCCCAGTCTCG

### Plasmid construction.

Plasmid pRV was first constructed by PCR amplification of the coding region of *ravA-viaA* from the E. coli MG1655 chromosomal DNA using the following primer pair: ravA UP BamHI/viaA DO HindIII (Table 2). The PCR product was then digested by BamHI and HindIII and cloned into the BamHI/HindIII linearized pTrc99A vector (38). The sequence of the inserted fragment was checked by DNA sequencing.

### Time-dependent killing assay.

Overnight cultures were diluted (1/100) and grown aerobically or anaerobically in specific medium as indicated in the figure legends at 37°C to an OD_600_ of 0.2. At this point (t0) antibiotic at the indicated concentration was added to the cells. At different incubation times, 100 μL of cells were diluted in sterile phosphate-buffered saline solution (PBS buffer), spotted on LB agar, and incubated at 37°C for 24 to 48 h. Cell survival was determined by counting CFU per mL (CFU/mL). The absolute CFU at time point 0 (used as the 100%) was ≈ 5 × 10^7^ CFU/mL. Survival rate in anaerobic conditions was performed in an anaerobic chamber (Coy and Jacomex Chambers). Materials (medium, tubes, plates, etc.) were all equilibrated in the anaerobic chamber for at least 18 h prior to use.

### MIC values determination.

The MICs were determined by the microdilution method in a 96-well plate according to the Clinical and Laboratory Standards Institute (CLSI) guideline. Briefly, serial dilutions of Gm in a 2-fold manner were done in 100 μL cation-adjusted Müller-Hinton or in LB supplemented or not with either glucose (0.2%), fumarate (10 mM), or nitrate (10 mM). E. coli inoculum was prepared by suspending colonies grown overnight on LB agar using 1×PBS to achieve a turbidity of 0.5 McFarland (1 × 10^8^ CFU/mL) and the final concentration of the inoculum in each well was around 5 × 10^5^ CFU/mL. The plates were incubated at 37°C for 18 h under aerobic or anaerobic conditions. MIC was defined as the lowest drug concentration that exhibited complete inhibition of microbial growth. All MICs were determined from at least three independent biological replicates.

### Gm uptake assays.

[^3^H]-Gm (20 μCi/mg; Hartmann Analytic Corp.) was added at the indicated final concentration and cultures were incubated at 37°C on a rotary shaker. At given times, 500 μL aliquots were removed and collected on a 0.45 μM-pore-size HAWP membrane filter (Millipore) pretreated with 1 mL of unlabeled Gm (250 μg/mL). Filters were subsequently washed with 10 mL of 3% NaCl, placed into counting vials, and dried for 30 min at 52°C, whereafter 8 mL of scintillation liquid were added and incubated overnight at room temperature. Vials were counted for 5 min. Gm uptake efficiency is expressed as total accumulation of Gm (ng) per 10^8^ cells.

### Competition experiment in batch culture.

The two strains tested were first grown separately overnight in M9 medium supplemented with casamino acids (0.1%). The cell density of each suspension was measured by OD_600nm_ reading and by CFU count. Each overnight culture containing approximately 3 × 10^8^ cells/mL was diluted 1/100-fold and mixed in a ratio of 1:1 to inoculate 25 mL of M9 supplemented with casamino acids (0.1%) (time 0 h) and incubated for 24 h at 37°C for a competitive growth. The coculture was diluted 1/100 in 25 mL of fresh M9 medium and grown for another 24 h at 37°C. The initial density of each strain was determined in the initial coculture (0 h) from CFU data by diluting and plating population samples onto LB agar and LB agar supplemented with appropriate antibiotic. Similarly, the final density of each strain was determined.

### Complex I enzymatic assay.

Cells grown in LB (100 mL) to an OD_600nm_ of 0.6 were harvested by centrifugation, washed once in 50 mM phosphate buffer pH 7.5, and suspended in 50 mM phosphate buffer pH 7.5 (6 mL), lysed using a French press, aliquoted, and frozen immediately in liquid nitrogen. Nuo activity was assayed at 30°C by adding thawed samples to 50 mM phosphate buffer pH 7.5 containing reduced nicotinamide hypoxanthine dinucleotide (deamino-NADH) (250 mM) as specific substrate, and by following A_340nm_. Protein concentration was determined by measuring the absorbance at A_280nm_ using a NanoDrop 2000 spectrophotometer.

## References

[1] Laxminarayan R, Duse A, Wattal C, Zaidi AKM, Wertheim HFL, Sumpradit N, Vlieghe E, Hara GL, Gould IM, Goossens H, Greko C, So AD, Bigdeli M, Tomson G, Woodhouse W, Ombaka E, Peralta AQ, Qamar FN, Mir F, Kariuki S, Bhutta ZA, Coates A, Bergstrom R, Wright GD, Brown ED, Cars O. 2013. Antibiotic resistance-the need for global solutions. Lancet Infect Dis 13:1057–1098. doi:10.1016/S1473-3099(13)70318-9. 24252483

[2] Blair JMA, Webber MA, Baylay AJ, Ogbolu DO, Piddock LJV. 2015. Molecular mechanisms of antibiotic resistance. Nat Rev Microbiol 13:42–51. doi:10.1038/nrmicro3380. 25435309

[3] Bryan LE, Kwan S. 1981. Mechanisms of aminoglycoside resistance of anaerobic bacteria and facultative bacteria grown anaerobically. J Antimicrob Chemother 8:1–8. doi:10.1093/jac/8.suppl_D.1. 7338485

[4] Schlessinger D. 1988. Failure of aminoglycoside antibiotics to kill anaerobic, low-ph, and resistant cultures. Clin Microb Rev 1:6. 10.1128/cmr.1.1.54 PMC358029 3060245

[5] Balaban NQ, Helaine S, Lewis K, Ackermann M, Aldridge B, Andersson DI, Brynildsen MP, Bumann D, Camilli A, Collins JJ, Dehio C, Fortune S, Ghigo J-M, Hardt W-D, Harms A, Heinemann M, Hung DT, Jenal U, Levin BR, Michiels J, Storz G, Tan M-W, Tenson T, Van Melderen L, Zinkernagel A. 2019. Definitions and guidelines for research on antibiotic persistence. Nat Rev Microbiol 17:441–448. doi:10.1038/s41579-019-0196-3. 30980069 PMC7136161

[6] Lobritz MA, Belenky P, Porter CBM, Gutierrez A, Yang JH, Schwarz EG, Dwyer DJ, Khalil AS, Collins JJ. 2015. Antibiotic efficacy is linked to bacterial cellular respiration. Proc Natl Acad Sci USA 112:8173–8180. doi:10.1073/pnas.1509743112. 26100898 PMC4500273

[7] Liu Y, Yang K, Zhang H, Jia Y, Wang Z. 2020. Combating Antibiotic Tolerance Through Activating Bacterial Metabolism. Front Microbiol 11:577564. doi:10.3389/fmicb.2020.577564. 33193198 PMC7642520

[8] Ezraty B, Vergnes A, Banzhaf M, Duverger Y, Huguenot A, Brochado AR, Su S-Y, Espinosa L, Loiseau L, Py B, Typas A, Barras F. 2013. Fe-S cluster biosynthesis controls uptake of aminoglycosides in a ROS-less death pathway. Science 340:1583–1587. doi:10.1126/science.1238328. 23812717

[9] Gerstel A, Beas JZ, Duverger Y, Bouveret E, Barras F, Py B. 2020. Oxidative stress antagonizes fluoroquinolone drug sensitivity via the SoxR-SUF Fe-S cluster homeostatic axis. PLoS Genet 16:e1009198. doi:10.1371/journal.pgen.1009198. 33137124 PMC7671543

[10] Chareyre S, Barras F, Mandin P. 2019. A small RNA controls bacterial sensitivity to gentamicin during iron starvation. PLoS Genet 15:e1008078. doi:10.1371/journal.pgen.1008078. 31009454 PMC6497325

[11] Taber HW, Mueller JP, Miller PF, Arrow AS. 1987. Bacterial uptake of aminoglycoside antibiotics. Microbiol Rev 51:439–457. doi:10.1128/mr.51.4.439-457.1987. 3325794 PMC373126

[12] Fraimow HS, Greenman JB, Leviton IM, Dougherty TJ, Miller MH. 1991. Tobramycin uptake in Escherichia coli is driven by either electrical potential or ATP. J Bacteriol 173:2800–2808. doi:10.1128/jb.173.9.2800-2808.1991. 2019557 PMC207860

[13] Herisse M, Duverger Y, Martin-Verstraete I, Barras F, Ezraty B. 2017. Silver potentiates aminoglycoside toxicity by enhancing their uptake. Mol Microbiol 105:115–126. doi:10.1111/mmi.13687. 28383153

[14] Bryan LE, Van Den Elzen HM. 1977. Effects of membrane-energy mutations and cations on streptomycin and gentamicin accumulation by bacteria: a model for entry of streptomycin and gentamicin in susceptible and resistant bacteria. Antimicrob Agents Chemother 12:163–177. doi:10.1128/AAC.12.2.163. 143238 PMC429880

[15] Alper MD, Ames BN. 1978. Transport of antibiotics and metabolite analogs by systems under cyclic AMP control: positive selection of Salmonella typhimurium cya and crp mutants. J Bacteriol 133:149–157. doi:10.1128/jb.133.1.149-157.1978. 201606 PMC221988

[16] Nichols WW, Young SN. 1985. Respiration-dependent uptake of dihydrostreptomycin by Escherichia coli. Its irreversible nature and lack of evidence for a uniport process. Biochem J 228:505–512. doi:10.1042/bj2280505. 2409962 PMC1145009

[17] Davis BD, Chen LL, Tai PC. 1986. Misread protein creates membrane channels: an essential step in the bactericidal action of aminoglycosides. Proc Natl Acad Sci USA 83:6164–6168. doi:10.1073/pnas.83.16.6164. 2426712 PMC386460

[18] Eisenberg ES, Mandel LJ, Kaback HR, Miller MH. 1984. Quantitative association between electrical potential across the cytoplasmic membrane and early gentamicin uptake and killing in Staphylococcus aureus. J Bacteriol 157:863–867. doi:10.1128/jb.157.3.863-867.1984. 6698939 PMC215339

[19] Unden G, Steinmetz PA, Degreif-Dünnwald P. 2014. The aerobic and anaerobic respiratory chain of Escherichia coli and Salmonella enterica: enzymes and energetics. EcoSal Plus 6. doi:10.1128/ecosalplus.ESP-0005-2013. 26442941

[20] Unden G, Bongaerts J. 1997. Alternative respiratory pathways of Escherichia coli: energetics and transcriptional regulation in response to electron acceptors. Biochim Biophys Acta 1320:217–234. doi:10.1016/s0005-2728(97)00034-0. 9230919

[21] Cole ST, Grundström T, Jaurin B, Robinson JJ, Weiner JH. 1982. Location and nucleotide sequence of frdB, the gene coding for the iron-sulphur protein subunit of the fumarate reductase of Escherichia coli. Eur J Biochem 126:211–216. doi:10.1111/j.1432-1033.1982.tb06768.x. 6751816

[22] Wong KS, Bhandari V, Janga SC, Houry WA. 2017. The RavA-ViaA chaperone-like system interacts with and modulates the activity of the fumarate reductase respiratory complex. J Mol Biol 429:324–344. doi:10.1016/j.jmb.2016.12.008. 27979649

[23] Girgis HS, Hottes AK, Tavazoie S. 2009. Genetic architecture of intrinsic antibiotic susceptibility. PLoS One 4:e5629. doi:10.1371/journal.pone.0005629. 19462005 PMC2680486

[24] Wong KS, Snider JD, Graham C, Greenblatt JF, Emili A, Babu M, Houry WA. 2014. The MoxR ATPase RavA and its cofactor ViaA interact with the NADH:Ubiquinone Oxidoreductase I in Escherichia coli. PLoS One 9:e85529. doi:10.1371/journal.pone.0085529. 24454883 PMC3893208

[25] Baharoglu Z, Babosan A, Mazel D. 2014. Identification of genes involved in low aminoglycoside-induced SOS response in Vibrio cholerae: a role for transcription stalling and Mfd helicase. Nucleic Acids Res 42:2366–2379. doi:10.1093/nar/gkt1259. 24319148 PMC3936754

[26] Kirkpatrick C, Maurer LM, Oyelakin NE, Yoncheva YN, Maurer R, Slonczewski JL. 2001. Acetate and formate stress: opposite responses in the proteome of Escherichia coli. J Bacteriol 183:6466–6477. doi:10.1128/JB.183.21.6466-6477.2001. 11591692 PMC100143

[27] Unden G. 1988. Differential roles for menaquinone and demethylmenaquinone in anaerobic electron transport of E. coli and their fnr-independent expression. Arch Microbiol 150:499–503. doi:10.1007/BF00422294. 2849923

[28] Pinske C, Jaroschinsky M, Linek S, Kelly C, Sargent F, Sawers G. 2015. Physiology and bioenergetics of [NiFe]-hydrogenase 2-catalyzed H 2 -consuming and H 2 -producing reactions in Escherichia coli. J Bacteriol 197:296–306. doi:10.1128/JB.02335-14. 25368299 PMC4272588

[29] Jones SA, Gibson T, Maltby RC, Chowdhury FZ, Stewart V, Cohen PS, Conway T. 2011. Anaerobic Respiration of Escherichia coli in the Mouse Intestine. Infect Immun 79:4218–4226. doi:10.1128/IAI.05395-11. 21825069 PMC3187261

[30] Laurinavichene TV, Tsygankov AA. 2001. H2 consumption by Escherichia coli coupled via hydrogenase 1 or hydrogenase 2 to different terminal electron acceptors. FEMS Microbiol Lett 202:121–124. doi:10.1111/j.1574-6968.2001.tb10790.x. 11506918

[31] Volbeda A, Darnault C, Parkin A, Sargent F, Armstrong FA, Fontecilla-Camps JC. 2013. Crystal structure of the O2-tolerant membrane-bound hydrogenase 1 from Escherichia coli in complex with its cognate cytochrome b. Structure 21:184–190. doi:10.1016/j.str.2012.11.010. 23260654

[32] Kaila VRI, Wikström M. 2021. Architecture of bacterial respiratory chains. Nat Rev Microbiol 19:319–330. doi:10.1038/s41579-020-00486-4. 33437024

[33] Pelosi L, Vo C-D-T, Abby SS, Loiseau L, Rascalou B, Hajj Chehade M, Faivre B, Goussé M, Chenal C, Touati N, Binet L, Cornu D, Fyfe CD, Fontecave M, Barras F, Lombard M, Pierrel F. 2019. Ubiquinone biosynthesis over the entire O2 range: characterization of a conserved O2-independent pathway. mBio 10:e01319-19. doi:10.1128/mBio.01319-19. 31289180 PMC6747719

[34] Jessop M, Felix J, Gutsche I. 2021. AAA+ ATPases: structural insertions under the magnifying glass. Curr Opin Struct Biol 66:119–128. doi:10.1016/j.sbi.2020.10.027. 33246198 PMC7973254

[35] Felix J, Bumba L, Liesche C, Fraudeau A, Rébeillé F, El Khoury JY, Huard K, Gallet B, Moriscot C, Kleman J-P, Duhoo Y, Jessop M, Kandiah E, Barras F, Jouhet J, Gutsche I. 2022. The AAA+ ATPase RavA and its binding partner ViaA modulate E. coli aminoglycoside sensitivity through interaction with the inner membrane. Nat Commun 13:5502. doi:10.1038/s41467-022-32992-9. 36127320 PMC9489729

[36] Lee J-Y, Tsolis RM, Bäumler AJ. 2022. The microbiome and gut homeostasis. Science 377:eabp9960. doi:10.1126/science.abp9960. 35771903

[37] Datsenko KA, Wanner BL. 2000. One-step inactivation of chromosomal genes in Escherichia coli K-12 using PCR products. Proc Natl Acad Sci USA 97:6640–6645. doi:10.1073/pnas.120163297. 10829079 PMC18686

[38] Amann E, Ochs B, Abel KJ. 1988. Tightly regulated tac promoter vectors useful for the expression of unfused and fused proteins in Escherichia coli. Gene 69:301–315. doi:10.1016/0378-1119(88)90440-4. 3069586

[39] Kazemzadeh K, Chehade MH, Hourdoir G, Brunet CD, Caspar Y, Loiseau L, Barras F, Pierrel F, Pelosi L. 2021. The biosynthetic pathway of ubiquinone contributes to pathogenicity of Francisella novicida. J Bacteriol 203:e00400-21. doi:10.1128/JB.00400-21. 34543102 PMC8570268

