# Supplementary material for: Bioenergetic State of Escherichia coli Controls Aminoglycoside Susceptibility
Source: mBio. 2023 Jan 10;14(1):e03302-22. doi: 10.1128/mbio.03302-22 (PMC9973319; doi:10.1128/mbio.03302-22)
Supplement: FIG S1 [file mbio.03302-22-s0001.docx]

**Bioenergetic state of *Escherichia coli* controls aminoglycoside susceptibility**

Jessica Y. El Khoury°, Jordi Zamarreño Beas°, Allison Huguenot, Béatrice Py, Frédéric Barras

°These authors contributed equally to this work and their names are listed in alphabetical order

**
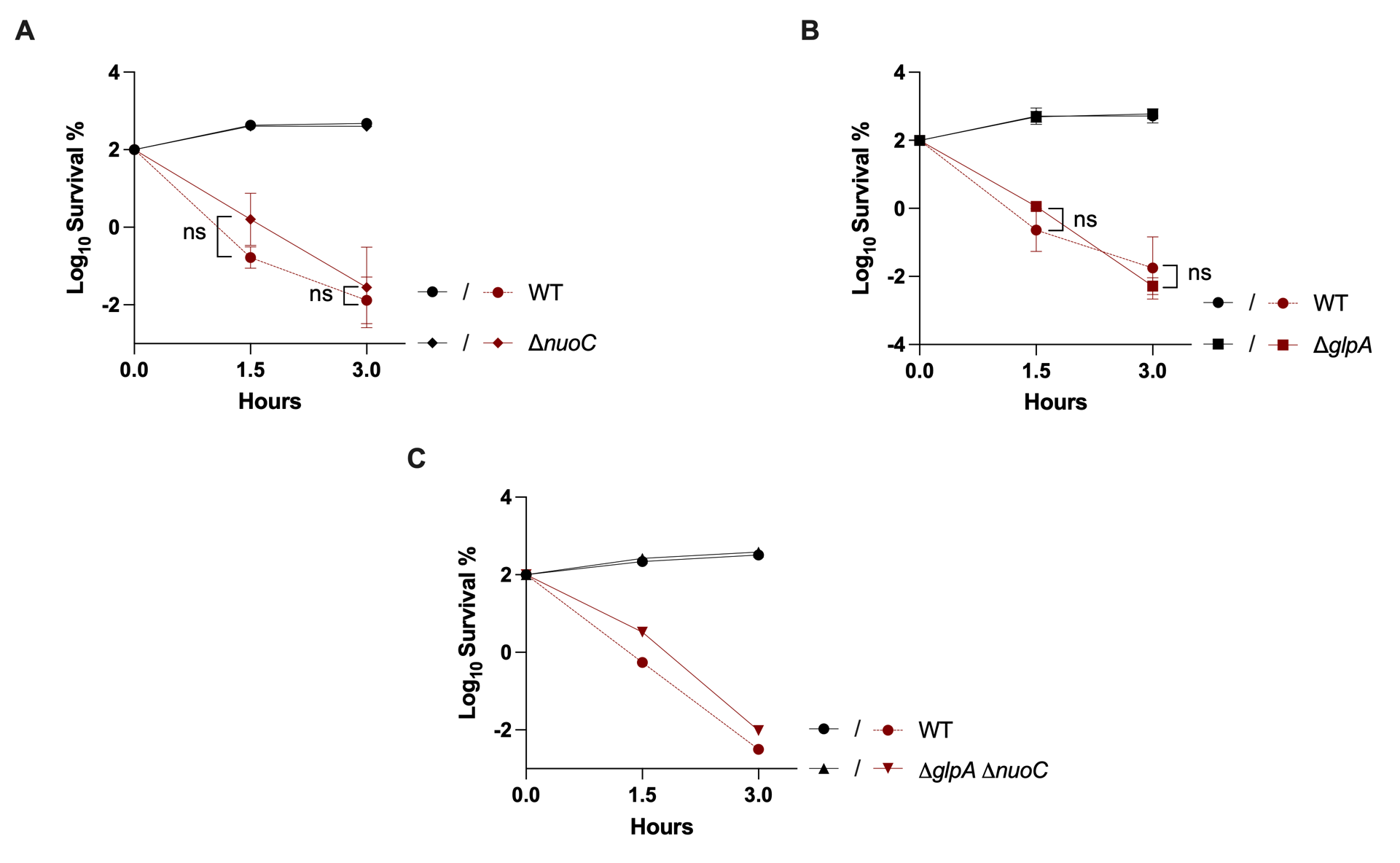
**

**S1 Fig. The Nuo complex and the GlpA complex are dispensable for the RavA/ViaA-dependent sensitization of *E. coli* to Gm under fumarate respiration.**

**(A, B, C)** Survival of WT (FBE051)**,** ∆*nuoC* (FBE1057), ∆*glpA* (FBE950) and ∆*glpA* ∆*nuoC* (FBE1055) strains after Gm treatment. Cells were grown in LB supplemented with fumarate at 10 mM **(A)** and glycerol at 0.2% **(B, C)** and then Gm was added at 16 µg/mL. The survival values after 1.5 and 3 hours of treatment are represented. Black and red lines are for untreated and Gm-treated bacteria, respectively. The lines of untreated cells are overlapping. Survival measured by CFU per mL, was normalized relative to time zero at which Gm was added (early log phase cells; ~5x10^7^ CFU/mL) and plotted as Log_10_ of % survival. For **(A)** and **(B)**, values are expressed as means of at least 3 biological replicates and error depict standard deviation. One-way ANOVA tests followed by Sidak’s multiple comparaison tests were performed to compare at each time point (1.5 and 3 hours) the treated WT to each of the treated mutant (ns = not significant).
