# Supplementary material for: Bioenergetic State of Escherichia coli Controls Aminoglycoside Susceptibility
Source: mBio. 2023 Jan 10;14(1):e03302-22. doi: 10.1128/mbio.03302-22 (PMC9973319; doi:10.1128/mbio.03302-22)
Supplement: FIG S2 [file mbio.03302-22-s0002.docx]

**Bioenergetic state of *Escherichia coli* controls aminoglycoside susceptibility**

Jessica Y. El Khoury°, Jordi Zamarreño Beas°, Allison Huguenot, Béatrice Py, Frédéric Barras

°These authors contributed equally to this work and their names are listed in alphabetical order

**
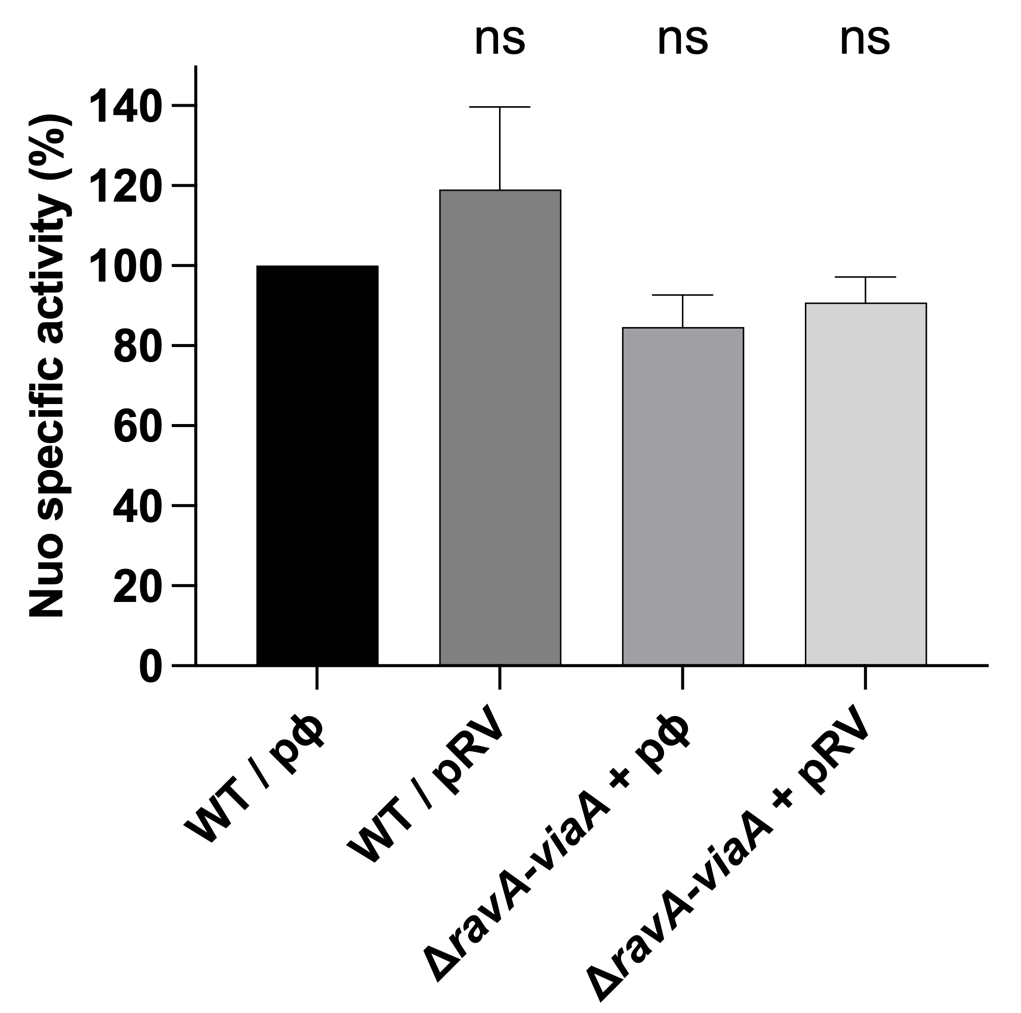
**

**S2 Fig. RavA-ViaA have no effect on Nuo activity.**

Nuo specific activity in the WT (FBE051) and the Δ*ravA-viaA* (FBE706) strains containing the plasmid carrying the *ravA-viaA* genes (pRV) or the corresponding empty vector (pØ). Nuo specific activity was measured in cells extracts using deamino-NADH as substrate. Values are expressed as means (n≥3) and error bars depict mean deviation. One-way ANOVA tests followed by Dunnett’s multiple comparaison tests were performed (ns = not significant). The 100 % corresponding to the activity in the WT strain is 127 nmol/min/mg protein.
